# Evaluation of a Clinic-Level Intervention to Increase Anxiety and Depression Screening in an Urban HIV Clinic

**DOI:** 10.1007/s10461-026-05102-9

**Published:** 2026-03-30

**Authors:** Daniela Zimmer, Erin M. Staab, Geetika Mehra, Mengqi Zhu, Jessica P. Ridgway, Jessica Schmitt, Melissa I. Franco-Galicia, Scott Hunter, Neda Laiteerapong

**Affiliations:** 1 Department of Medicine, University of Chicago, Chicago, IL, USA; 2 Chicago Center for HIV Elimination, Department of Medicine, University of Chicago, Chicago, IL, USA; 3 WCG Clinical Endpoint Solutions, Princeton, NJ, USA; 4 Department of Psychiatry and Behavioral Neuroscience, University of Chicago, 5841 S Maryland Ave, Chicago, IL 60637, USA

**Keywords:** Depression, Anxiety, Screening, HIV, Population health

## Abstract

Anxiety and depression are common in people living with HIV and associated with low antiretroviral treatment adherence, elevated HIV viral load, and increased mortality. Yet, anxiety and depression are underdiagnosed and undertreated in this population. Implementing a system for routine anxiety and depression screening may overcome barriers to diagnosis. To improve the identification and management of anxiety and depression within an HIV clinic, we engaged HIV clinicians in the design of the workflow, created electronic health record reminders for screening, and trained medical assistants to deliver the screening questions at clinic visits. We evaluated this 24-month quality improvement project using electronic health record data and clinician surveys. From November 2020 to October 2022, 747 patients had 1166 appointments during which anxiety and/or depression screening was due. During year one, anxiety and depression screening were completed at 75% (311/416) and 77% (362/469) of eligible encounters, respectively. During year two, screening was completed at 85% of encounters for anxiety (425/502) and depression (446/524). On average, anxiety screening increased by 2.2% per month (t = 4.24, *p* < 0.001), and depression screening increased by 1.2% per month (t = 2.82, *p* = 0.01) after intervention implementation. Patients who screened positive received follow-up. Clinician satisfaction with screening processes increased from baseline to 6 months. Findings suggest that screening for anxiety and depression can improve detection and management in an HIV clinic, while also being acceptable to HIV clinicians.

## Introduction

Nearly half of all persons living with HIV experience some form of a mental health disorder, primarily anxiety and depression [1, 2]. High rates of anxiety and depression may be due in part to societal discrimination, stigma, and negative self-perceptions [3]. Anxiety and depression contribute to negative outcomes, including lower engagement in healthcare [4], lower adherence to treatment plans [5–10], and increased participation in condomless sex [11, 12]. Yet, anxiety and depression are underdiagnosed in people with HIV [1].

Screening for mental health conditions is recommended by the Department of Health and Human Services and the Health Resources and Services Administration Ryan White HIV/AIDS Program [13]. Despite this recommendation, clinic barriers such as high workload, lack of time, lack of training, and inadequate referral resources often prevent the timely assessment of mental health conditions among people living with HIV [14–17]. Effective strategies for increasing screening in this population are needed [16, 18–20].

Little research has demonstrated how to improve screening for mental health conditions in people living with HIV [14, 21, 22]. Currently, HIV clinicians largely determine anxiety and depression through conversations with their patients without systematic screening methods or use of validated tools [3, 23–25]. Adopting brief screeners in HIV care could improve detection [26, 27]. Research has found that anxiety and depression symptoms measured using brief screeners correspond with estimates of anxiety and depression prevalence among people living with HIV [7, 17, 21, 28–32].

Developing anxiety and depression screening interventions is important for HIV treatment adherence, patient retention, and management [14, 32–35]. This project aimed to increase anxiety and depression screening and management at an urban academic HIV clinic located on the South Side of Chicago by developing and implementing system-level changes to clinic workflow.

## Methods

### Project Design

The Patient Outcomes Reporting for Timely Assessment of Life with HIV and Anxiety Disorders (PORTAL HIV-A) project was a 24-month quality improvement project (November 2020-October 2022) to implement anxiety and depression screening and management in an HIV clinic. According to institutional policy, this was considered a quality improvement project, as anxiety and depression screening are recommended components of routine care. Additionally, a protocol for surveying HIV clinicians was reviewed and approved by the Institutional Review Board.

### Setting

At the time of the intervention, 24 HIV clinicians (physicians and advanced practice nurses) and two medical social workers were providing care in the clinic. Usual care in the clinic before the intervention consisted of clinicians identifying patients with anxiety and depression using clinical gestalt (e.g., if a patient explicitly reported symptoms consistent with anxiety or depression). Standardized anxiety and depression questionnaires were available but used inconsistently [36]. Patients who expressed anxiety or depression symptoms were typically referred to a medical social worker to access community behavioral health resources.

### Intervention

We met with HIV clinicians in July 2020 to discuss potential strategies for implementing anxiety and depression screening in the HIV clinic. Clinicians reviewed the screening protocol that had been developed in primary care at the institution and chose to adopt that strategy in its entirety. Clinical decision support tools developed for the general primary care population were reviewed by the HIV pharmacist to ensure that medication recommendations would be applicable to patients with HIV.

The clinic-level intervention previously used in primary care has been described [19]. In brief, the electronic health record (EHR, Epic) was updated such that annual electronic reminders (best practice advisories) would prompt anxiety and depression screening during clinic visits for patients with HIV. Best practice advisories were visible to clinicians and medical assistants and linked to validated questionnaires to facilitate screening

The questionnaires used were the Generalized Anxiety Disorder screener-7 (GAD-7) for anxiety and the Patient Health Questionnaire (PHQ-9) for depression [37, 38]. If a patient did not have a history of anxiety or depression documented in the EHR, then the first 2 questions of the GAD-7 or PHQ-9 was used as an initial screener; for patients who scored 3 or greater on these first two items, the screening form would automatically expand to the full GAD-7 or PHQ-9. If a patient had a history of anxiety or depression documented in the EHR or a recent positive screen (score of 10 or greater), the best practice advisory was linked to the full GAD-7 or PHQ-9, rather than the initial 2 questions.

When a patient screened positive, an alert would display in the patient’s chart to notify the clinician of the result so that they could address it during the visit; this alert included a link to a smart order set that could be used to prescribe medications and place referrals. Medical assistants were trained to administer questionnaires either verbally or on paper, depending on patient comfort and how much time they had available. Patients with clinic appointments were considered due for screening if they had not been screened within the last year.

### Evaluation

The intervention was evaluated using a combination of EHR and clinician survey data. For the EHR data, the primary outcomes were rates of anxiety and depression screening at HIV clinic visits. As secondary outcomes, we assessed management of anxiety and depression among those who screened positive. Management was defined as inclusion of positive screening results in the HIV clinician’s assessment and plan for the visit and/or one or more of the following within 12 months: subsequent visit with HIV clinician in which anxiety/depression was addressed, visit with HIV clinic social worker, referral to mental health specialist or primary care clinician, or psychotropic medication initiation or titration. The institution’s Center for Research Informatics provided data on screening. Project team members conducted a chart review to collect data on anxiety and depression management; data were extracted by two people and discrepancies were resolved via discussion.

The clinician survey was used to understand changes in attitudes, clinical practice, and confidence in managing anxiety and depression. A 37-question survey was developed by the authors with items adapted from prior work in primary care [39]. The survey was sent to HIV clinicians (all attending and fellow physicians and advanced practice nurses, except one physician who was study co-principal investigator) at implementation (December 2020) and after six months (July 2021). Attitudes about anxiety and depression screening were measured using a Likert type scale from 1 to 5, with one indicating strongly disagree and five indicating strongly agree. Participants were asked about their practice of screening for anxiety and depression, satisfaction with the clinic’s screening practices, and level of comfort diagnosing, treating, and monitoring symptoms of anxiety and depression. Survey invitations were sent via email and responses were collected in REDCap. Clinicians were reminded about the survey at clinic meetings and via email. Survey completion served as a proxy for consent.

### Analysis

Descriptive analysis was used to summarize the demographic information of patients seen in the clinic and the clinician survey data. The screening rate was calculated as the percentage of visits at which screening was due that had a completed screen. A logistic growth model $y=\frac{L}{1+e^{-k(t-t_{0})}}$ was used to assess the change in monthly screening rates. In the logistic growth model, *L* represents the maximum value that the screening rate could asymptotically reach, k represents the steepness or rate of change of the logistic curve, and *t*_0_ is the estimated value of the month at which the screening rate changes most rapidly. The parameters were estimated using nonlinear least squares and tested whether they were significantly different from 0 using t-statistic. Analysis was performed using R version 4.2.0 (R Foundation for Statistical Computing; Vienna, Austria, 2022). Survey analyses were a cross-sectional comparison over time. Responses were dichotomized (1=strongly agree/agree, 0=undecided/disagree/strongly disagree), and chi-square and Fisher’s exact tests were used to determine whether there was a change in opinions on anxiety and depression screening from initial to follow-up survey using SAS [40]. A p-value of < 0.05 was considered significant.

## Results

### Screening Rates

From November 2020 to October 2022, there were 747 unique patients with HIV who attended 1166 clinic visits during which screening for anxiety and/or depression was due. Patients’ average age was 46 years (SD = 15), with 31% (231) identifying as female and 84% (626) identifying as non-Hispanic Black (Table 1).

**Fig. 1:**
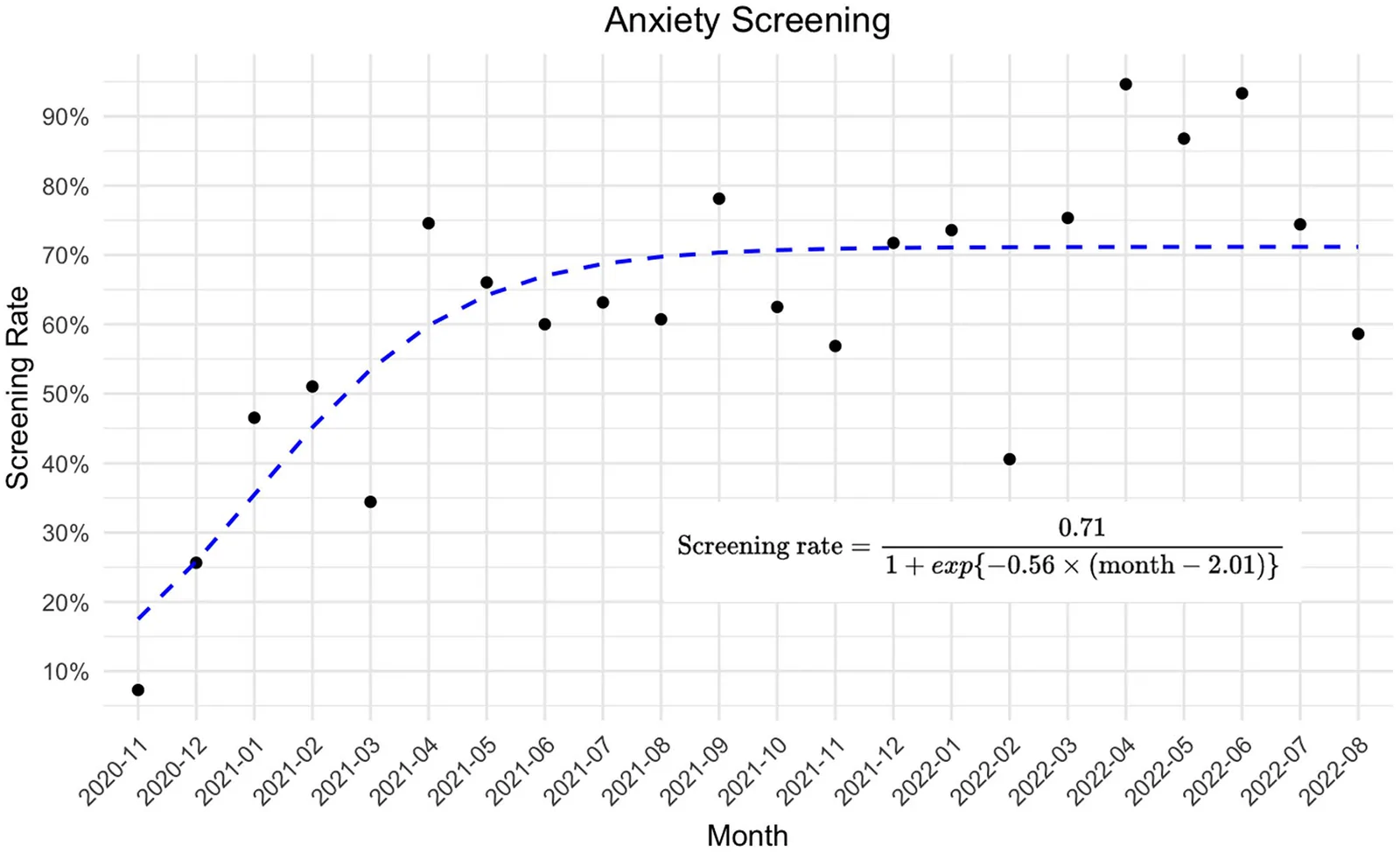
Monthly anxiety screening rates in HIV clinic, November 2020–August 2022

**Fig. 2:**
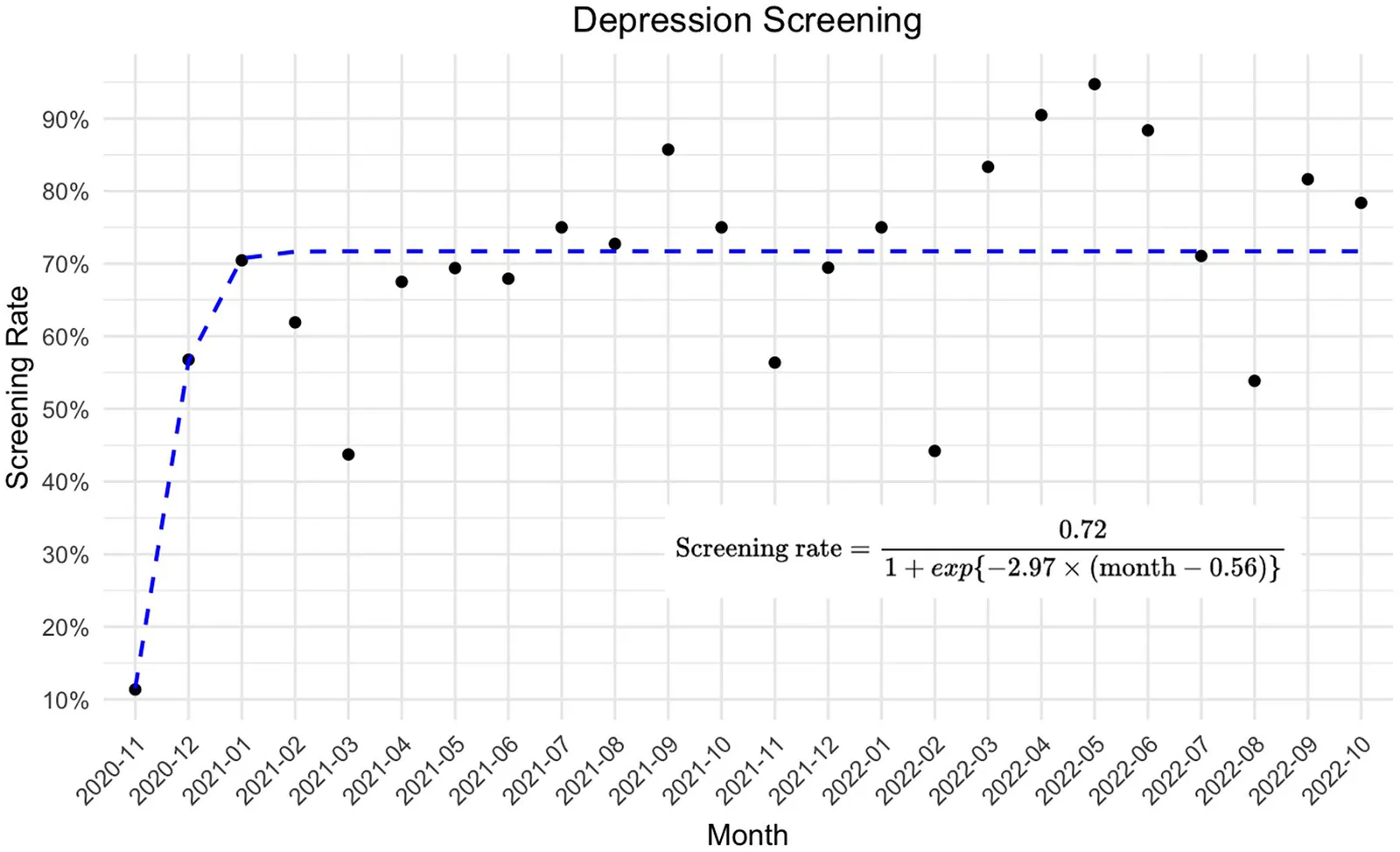
Monthly depression screening rates in HIV clinic, November 2020–October 2022

Anxiety screening was completed at 75% (311/416) of visits at which it was due in the first year of implementation and 85% (425/502) in year two. Depression screening was completed at 77% (362/469) of visits in year one and 85% (446/524) of visits in year two. The logistic growth model showed that the anxiety screening rate started at 7% of visits and increased most rapidly around month 2. The steepness parameter (k = 0.56 (t = 2.27, *p* = 0.04)) suggests that the overall screening rate transitioned gradually and significantly over time (Fig. 1). Similarly, there was a rapid increase in depression screening within the first month after intervention started; however, this increase stopped after month 3 and the steepness parameter (k = 2.97 (t = 1.65, *p* = 0.11)) indicates that the screening rate increased relatively quickly, but the rapid growth did not last (Fig. 2).

Overall, 6% (46/747) of eligible patients screened positive for anxiety and 6% (43/747) screened positive for depression. Results were addressed for all patients who screened positive either by the HIV clinician at the same visit and/or through referral, follow-up, or medication within 12 months. About half of patients who screened positive had management by their HIV clinician, about half were referred to a mental health specialist, about a third started or adjusted a psychotropic medication, and about a third had a visit with the HIV clinic social worker (Table 2).

### Clinician Survey

Response rates were 57% (13/23) for the baseline survey and 52% (12/23) for the 6-month follow-up survey. Sixteen clinicians completed at least one survey (70%; 16/23). At baseline and 6 months, all clinicians agreed that patients with HIV should be screened for depression at least once a year and that screening for depression would help them provide better care (Table 3). Agreement with these statements was lower for anxiety but increased at 6 months. For example, 77% of clinicians agreed that patients with HIV should be screened for anxiety at least once a year at baseline, and this rate increased to 100% at 6 months. Most clinicians agreed that monitoring symptoms of anxiety and depression was an essential part of HIV care.

At baseline, there was low satisfaction with the clinic’s screening process and low agreement that patients were screened annually for anxiety and depression. At 6 months, there was an increase in satisfaction with the HIV clinic’s screening process for anxiety (1 (8%) vs. 6 (50%), *p* = 0.03) and depression (0 (0%) vs. 5 (42%), *p* = 0.02). Also, clinicians were more likely to agree that the clinic screened for depression annually (2 (15%) vs. 7 (58%), *p* = 0.04). Similarly, more clinicians agreed that the clinic screened patients with HIV for anxiety annually, but this was not significant (1 (8%) vs. 5 (42%), *p* = 0.07). Less than half of clinicians thought that reviewing screening results was burdensome or reported that they did not have time to ask about anxiety and depression symptoms. However, most clinicians did not feel confident in their ability to treat anxiety and depression.

## Discussion

This two-year quality improvement project adds important information on how to effectively implement anxiety and depression screening in HIV clinics. After adopting clinical workflows from primary care [19, 20], there was a significant increase in depression and anxiety screening in patients with HIV at this clinic. Screening during clinic visits identified patients with active anxiety or depression symptoms, and results were addressed either in the HIV clinic or via referral to primary care or mental health. Overall, HIV clinicians believed that depression and anxiety screening and management was important and essential to HIV care, and their satisfaction increased with implementation of the new workflows. Still, the majority were not confident in their ability to treat these conditions themselves.

Previous studies have targeted clinician education and behaviors to increase anxiety and depression screening without the addition of best practice advisories or utilizing other clinic staff to assist with screening [41, 42]. Studies recommend including staff outside of the clinician in the workflow to increase mental health screening uptake [19, 20, 43]. Additionally, access to behavioral health management has been a challenge in other screening interventions; availability of resources impacts clinicians’ desire to implement standardized anxiety and depression screening [15, 44].

Relying on clinician training without larger infrastructural support (treatment referrals, mental health screening assistance) does not result in increased clinician confidence for anxiety or depression diagnosis or treatment [14, 20, 45, 46]. Factors such as patient-clinician relationships, clinic demographics, and the implementation of staff-led anxiety and depression screening protocols impact clinician confidence [19, 20, 47, 48]. Clinicians at the HIV clinic reported a significant increase in their satisfaction with the process of screening after adopting staff-led anxiety and depression screening, consistent with the literature [20, 46, 49–51]; however, improvements are still needed since about half of respondents were not satisfied. Additional efforts, such as clear pathways for treatment, are likely needed.

There are several limitations to this work. This observational project was conducted in a single HIV clinic in a large academic hospital, and results may be different at other settings. We were able to leverage tools and support from other clinics that had already identified effective strategies for screening, which may not be available to all HIV clinics. Additionally, our surveys were cross-sectional with moderate response rates, so results are subject to non-response and composition bias.

This project found an increase in anxiety and depression screening after engaging HIV clinicians in the design of new workflows, using knowledge and resources gained from primary care, and implementing annual EHR reminders for screening by medical assistants at clinic visits. This project also demonstrated that HIV clinicians understand the importance of mental health screening and management but experience common barriers like lack of time and confidence to treat mental health disorders. Future work should include clear pathways for the treatment of anxiety and depression and assess sustainment of the intervention.

## Figures and Tables

**Table 1:** Characteristics of patients due for anxiety/depression screening in HIV clinic, November 2020–October 2022 (*N* = 747)

	Mean*/n*	SD/%
Age, years	46	15
*Sex*		
Male	516	69
Female	231	31
*Race*		
Hispanic	30	4
Non-Hispanic Black	626	84
Non-Hispanic White	69	9
Non-Hispanic Asian	5	1
Other or Unknown	17	2
*Insurance*		
Medicaid	317	42
Medicare	86	12
Medicare/Medicaid	88	12
Private	234	31
Unknown	22	3

**Table 2:** Management of depression and anxiety within 12 months of positive screen

	Anxiety, *n* (%)	Depression, *n* (%)
Any management documented	46 (100%)	43 (100%)
Positive screening results included in HIV clinician assessment and plan for visit	23 (50%)	26 (60%)
Follow-up in HIV clinic, referral, and/or medication within 12 months of positive screen	28 (61%)	30 (70%)
Follow-up in HIV clinic	14 (30%)	17 (40%)
Visit with HIV clinic social worker	13 (28%)	13 (30%)
Subsequent visit with HIV clinician in which anxiety/depression was addressed	4 (9%)	9 (21%)
Referral to another provider	18 (39%)	26 (60%)
Referral to mental health specialist	18 (39%)	26 (60%)
Referral to primary care clinician	1 (2%)	1 (2%)
Psychotropic medication initiation or titration	15 (33%)	14 (33%)

**Table 3:** Clinician perspectives on anxiety and depression screening in the HIV clinic at baseline and 6 months

	Anxiety			Depression		
	Baseline *n* (%) agree/strongly agree	6 Months *n* (%) agree/strongly agree	*p*-value	Baseline *n* (%) agree/strongly agree	6 Months *n* (%) agree/strongly agree	*p*-value
*General attitudes*						
Patients with HIV should be screened at least annually	10 (77)	12 (100)	Fisher’s exact *p* = 0.22	13 (100)	12 (100)	–
Screening helps me provide better care for my patients	11 (85)	11 (92)	Fisher’s exact *p* = 1.00	13 (100)	12 (100)	–
Monitoring symptoms is an essential part of HIV care	9 (69)	11 (92)	Fisher’s exact *p* = 0.32	13 (100)	10 (83)	Fisher’s exact *p* = 0.22
*Attitudes about clinic screening and management*						
I am satisfied with our clinic’s screening process	1 (8)	6 (50)	Fisher’s exact *p* = 0.03	0 (0)	5 (42)	Fisher’s exact *p* = 0.02
Patients in our clinic are screened at least annually	1 (8)	5 (42)	Fisher’s exact *p* = 0.07	2 (15)	7 (58)	Fisher’s exact *p* = 0.04
Reviewing screening results will be burdensome	4 (31)	5 (42)	Fisher’s exact *p* = 0.69	2 (15)	2 (17)	Fisher’s exact *p* = 1.00
Our clinic does a good job treating	0 (0)	2 (17)	Fisher’s exact *p* = 0.22	2 (15)	3 (25)	Fisher’s exact *p* = 0.65
*Current practices and confidence*						
I do not have time to ask about symptoms during visits	4 (31)	4 (33)	Fisher’s exact *p* = 1.00	2 (15)	3 (25)	Fisher’s exact *p* = 0.65
I do not have time to treat	8 (62)	7 (58)	Fisher’s exact *p* = 1.00	5 (38)	9 (75)	χ^2^ (1, 25) = 3.38 *p* = 0.07
I feel confident in my ability to diagnose	5 (38)	7 (58)	χ^2^ (1, 25) = 0.99 *p* = 0.32	6 (46)	8 (67)	χ^2^ (1, 25) = 1.06 *p* = 0.30
I feel confident in my ability to treat	1 (8)	1 (8)	Fisher’s exact *p* = 1.00	3 (23)	4 (33)	Fisher’s exact *p* = 0.67

## Data Availability

The datasets analyzed during the current study are not publicly available because they were gathered as part of usual clinical care and patient consent for data sharing was not provided.
